# Assessment of the relationship between subclinical hypothyroidism and blood lipid profile: reliable or not?

**DOI:** 10.1186/s12944-022-01749-0

**Published:** 2022-12-13

**Authors:** Yi Luo, Fei Wu, Zhen Huang, Yan Gong, Yansong Zheng

**Affiliations:** 1 The Department of Health Medicine, Second Medical Center & National Clinical Research Center for Geriatric Diseases, Chinese People’s Liberation Army General Hospital, 28# Fuxing Road, Haidian District, Beijing, 100853 China; 2 Health Management Department of China Health Promotion Foundation, Beijing, China; 3 Health Management Center of Nanning First People’s Hospital, Nanning, China

**Keywords:** Subclinical hypothyroidism, Lipid metabolism, Thyroid-associated hormones, Diagnosis, Criteria

## Abstract

**Background:**

The relationship between subclinical hypothyroidism (SCH) and blood lipid metabolism is controversial. This study is intended to evaluate the relationship between SCH and blood lipid profiles using well defined diagnostic criteria.

**Methods:**

Data from 11,512 physical examinees in our hospital who had finished two tests with an interval of at least 3 months were analyzed, including 685 cases of SCH as stipulated in this study. In addition to common physical examination indicators, other parameters such as thyroid function indices and blood lipids were measured twice with an interval of at least 3 months. Subjects were diagnosed with SCH only when their levels of TT3, TT4, FT3 and FT4 were normal and TSH levels were increased on both tests, which meant these subjects were in a state of SCH for at least 3 months. The results of blood lipids for the second time were analyzed.

**Results:**

Statistically significant differences were found in age, sex, BMI, hemoglobin, total cholesterol (TC), low density lipoprotein (LDL-C), high density lipoprotein (HDL-C), and BFP between the SCH and control groups (*P* < 0.001). However, there were no statistically significant differences in age, sex, blood pressure, blood lipids, blood glucose or BMI between patients with mild SCH and those with severe SCH (*P* > 0.05). After balancing the age and sex ratio, no factors were confirmed to be statistically significant independent factors of SCH. None of the parameters showed statistically significant differences between patients with mild SCH and those with severe SCH (*P* > 0.05).

**Conclusion:**

After defining rigorous criteria for the diagnosis of SCH, no definite association between SCH and TC, LDL-C or HDL-C was confirmed in this study. SCH may have no relationship to the most concerning blood lipid profile.

## Introduction

Subclinical hypothyroidism (SCH) is diagnosed when serum TSH is high and circulating thyroid hormones are within the reference range [1–3]. SCH may be a transient condition because these levels can return to normal or develop into overt hypothyroidism. According to established guidelines, SCH should not be diagnosed unless its manifestations are confirmed in two tests that are at least 3 months apart [3]. However, these guidelines rarely have been followed, and the criteria for SCH, and the diagnostic levels of TSH are inconsistent. There are significant differences in the way in which SCH is diagnosed, leading to the heterogeneity of SCH cases. These discrepancies have resulted in controversies regarding the diagnosis and clinical significance of SCH [4].

Previous studies showed that the diet structure of human had a strong correlation with thyroid diseases such as SCH [5]. And diet has a close relationship with various chronic diseases such as dyslipidemia [6], diabetes [7] and hypertension [8]. And the fact that dyslipidemia is a risk factor for cardio cerebrovascular related diseases is already indisputable. So, is there a correlation between SCH and lipid profiles?

There is mounting evidence that SCH can influence a variety of diseases such as overt hypothyroidism, thyroid malignancy, coronary heart disease [9], hypertension, ischemic cerebrovascular disease, metabolic syndrome, fractures [10], osteoporosis, abortion [11] and premature delivery [12]. It has been found in related studies that the levels of serum triglyceride (TG), total cholesterol (TC), low-density lipoprotein-cholesterol (LDL-C), and apolipoprotein (a) in patients with SCH are variably increased compared to those in the normal population [13]. Some studies found that the higher the thyroid-stimulating hormone (TSH), the more pronounced the lipid metabolism disorder. When serum TSH was increased by 1.0 mmol/L, TC levels increased correspondingly by 0.09-0.16 mmol/L [14, 15]. It has also been found that after clinical treatment, blood lipids decrease, especially lipoprotein A [16]. However, the prevalence and biochemical characteristics of SCH are likely to vary according to demographics and nutritional habits [17]. Moreover, most studies have shown that SCH interventions have not been able to correct dyslipidemia [18].

An explanation for these conflicting results among these cross-sectional studies is poor control in potential confounding factors, such as sex, age, race, dietary habit, which may be associated with lipid levels. SCH may be a transient state, and its effects on blood lipid metabolism are controversial. Moreover, blood lipid changes observed after intervention for SCH have not been defined. Given the lack of specific diagnostic criteria and treatment guidelines for SCH, we hypothesized that there was no substantial correlation between SCH and blood lipids. Thus, in this study, we developed a rigorous standard for diagnosing SCH by combining different diagnostic standards used in literature and defined the effect of SCH on lipid profile.

## Materials and methods

### Study design and participants

**Fig. 1:**
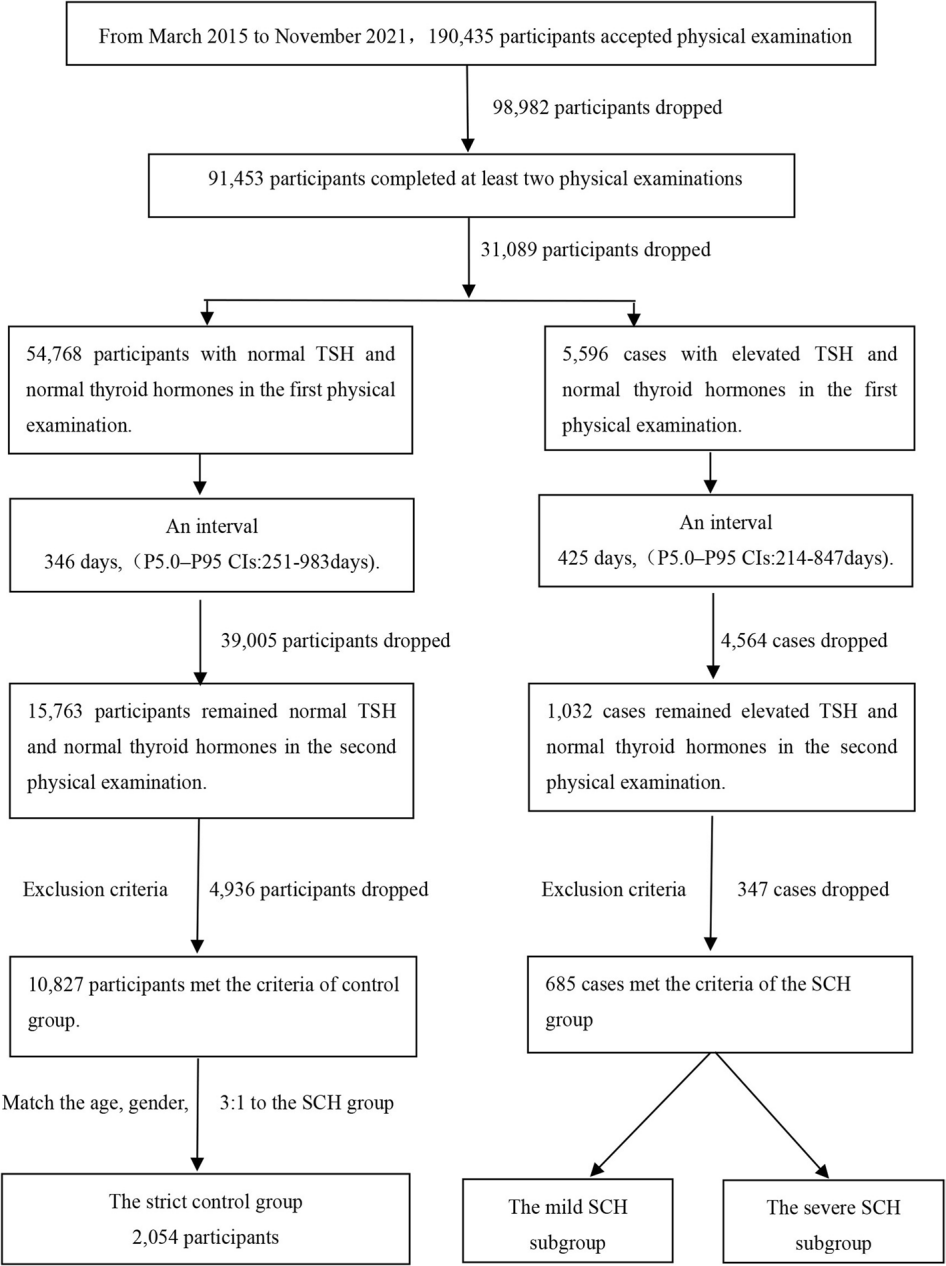
Flow diagram of enrolled participants. List of Abbreviations: TSH: thyroid-stimulating hormone; CI: Confidence Intervals; SCH: subclinical hypothyroidism

We used data from physical examinees in Chinese PLA General Hospital between March 2015 and November 2021. By using the population of annual physical examinations to form a queue, we compared the difference in physical examination results between patients with a definite diagnosis of SCH and the non-SCH population. Inclusion criteria were as follows: patients aged 18 or over who had undergone thyroid function tests and met the diagnostic criteria for SCH or were considered as having normal levels. Exclusion criteria included those with incomplete demographic information, incomplete results in five tests of thyroid function, the lack of repeat tests at least 3 months apart, the use of thyroxine or antithyroid drugs at baseline, medications affecting lipid metabolism, actively taking amiodarone, estrogen, glucocorticoids [19] or dopamine [20], pregnancy, and complications with other diseases affecting thyroid function or TSH hormone secretion such as malignant tumors. In total, 190,435 participants were enrolled in the study. In the control group, 54,768 participants had normal TSH, total thyroxine (TT4), total triiodothyronine (TT3), free thyroxine (FT4), free triiodothyronine (FT3), thyroglobulin antibody (TG-Ab), thyroid peroxidase antibody (TPO-Ab) in the first physical examination, and 15,763 participants remained normal in the second physical examination within a median interval of 346 days, and 4936 participants were excluded according to exclusion criteria. A total of 5596 patients were diagnosed with SCH in the first physical examination, and 1032 patients remained SCH state after 425 days (median);347 patients were excluded according to the exclusion criteria. Finally, 11,512 participants remained for the main analysis. The strict control group was randomly selected from the 10,827 participants in the normal control group (*N* = 2054), matching the age and sex composition ratio in the SCH group (Fig. 1). All procedures in this study complied with the guidelines of the Helsinki Declaration on Human Experimentation. The study protocol was approved by the Ethical Committee of the PLA General Hospital, and written, informed consent was obtained from all participants.

### Measurements

Age was calculated based on the birth date on an ID card, and the sex of the subjects was recorded. Questionnaires were conducted to determine history of hypertension, thyroid nodules, fatty liver, and malignant tumors. Thyroid nodules and fatty liver were diagnosed using standard ultrasonic methods. Smoking was defined as consuming ≥10 cigarettes per day for at least 1 year, which was consistent with the World Health Organization (WHO) definition of smoking [21]. In physical examinations we measured height, weight, and blood pressure, whereas laboratory tests included as analysis of blood lipids and blood glucose. Body mass was measured by a body composition analyzer, and body mass index (BMI = weight/height^2^ kg/m^2^) was calculated. All patients were dressed in uniform, loose physical examination clothing for measurement, had fasted for 8 – 12 h, and had their fasting blood glucose detected. In accordance with the quality control and testing criteria of the Clinical Laboratory Department of Chinese PLA General Hospital [22, 23], Roche’s diagnostic reagents were used to determine the levels of total cholesterol (TC), triglycerides (TG), high-density lipoprotein cholesterol (HDL-C), low-density lipoprotein cholesterol (LDL-C), fasting blood glucose (FBG), glycosylated hemoglobin (HbA1c), uric acid (UA), homocysteine (Hcy), TT4, TT3, FT4, FT3, TG-Ab, TPO-Ab and TSH (high sensitivity immunoassay) using an ACS180 automatic chemiluminescence immunoassay analyzer. Assays had an intrabatch difference of < 5% and intrabatch difference of < 10%. The normal reference range, was: 66-181 nmol/L for TT4, 1.3-3.1 nmol/L for TT3, 3.1-6.8 pmol/L for FT3, 12-22 pmol/L for FT4, < 115 IU/ml for TG- Ab, < 34 IU/ml for TPO-Ab, and 0.1 < TSH < 4.5 mIU/L. Physical examinations were performed twice, at least 3 months apart. The blood lipid profiles discussed in this study mainly include TC, TG, HDL-C and LDL-C. The results of blood lipids for the second time were analyzed.

### Grouping and criteria

According to China’s guidelines for diagnosing and treating adult hypothyroidism (2017 edition) [24], the subjects were divided into two groups. One was the control group with normal TSH and normal TT3, TT4, FT3, FT4, TG-Ab, and TPO-Ab in both physical examinations, and the other was the SCH group with elevated TSH and normal TT3, TT4, FT3, and FT4 on both physical tests. The SCH group was again divided into two subgroups: mild SCH subgroup (4.5 < TSH < 10 mIU/L) and severe SCH subgroup (TSH ≥ 10 mIU/L).

### Statistical analyses

Data were collected and analyzed using Stata software (version 11.0) (STATA, College Station, TX). Descriptive statistics were expressed as the mean ± standard deviation or median and 95% confidence intervals (CI), and categorical variables were described by frequencies and percentages. Comparisons between characteristics of the different groups were made using t-tests, χ2-tests, rank-sum tests or Kruskal-Wallis rank tests, because some indicators could not fully conform to the normal distribution. Multivariate logistic regression analysis was conducted by taking SCH as the dependent variable and other indices as independent variables. *P* < 0.05 indicates a statistically significant difference.

## Results

### Participant characteristics

Of the 11,512 subjects finally enrolled in the study, 8234 were males (71.53%) and 3378 were females (28.47%). The average age was 49.05 ± 8.88 years. Subjects who underwent physical examination came from 34 provinces, autonomous regions, municipalities or special administrative regions across China. The median interval between the two tests of those in the SCH group was 425 days, (95th confidence interval (CI) (P_2.5_–P_97.5_ *CI*:214-847 days). The median interval between the two tests of those in the control group was 346 days, 95th CI (P_2.5_–P_97.5_ *CI*:251-983 days).

### Distribution characteristics of patients with SCH

The comparison of clinical indices between subjects with SCH and the control group is shown in Table 1. The rank-sum tests showed statistically significant differences in BMI, hemoglobin, TC, LDL-C, HDL-C, FBG, Hcy, and UA between the two groups (*P* < 0.05). Multivariate logistic regression analysis was conducted by taking SCH as the dependent variable and other indices as independent variables. Finally, it was found that sex (*OR* = 0.419, *Z* = 9.52, *P* < 0.001, 95% *CI*:0.350-0.501), age (*OR* = 1.020, *Z* = 4.43, *P* < 0.001, 95% *CI*: 1.011-1.029), TC (*OR* = 1.155, *Z* = 3.31, *P = 0.001*, 95% *CI*: 1.060-1.258) and BMI (*OR* = 1.019, *Z* = 2.15, *P = 0.031*, 95% *CI*: 1.002-1.036) were the significant influencing factors of SCH. Other indices were not independent risk factors for SCH.

**Table 1:** Comparison of participant characteristics between the SCH group and the control group

	Total (***N*** = 11,512)	Control group (***N*** = 10,827)	SCH group(***N*** = 685)	Statistical value, ***P*** value
Age(years)	49.05 ± 8.88	48.94 ± 8.83	50.82 ± 9.42	*t* = 5.398, *P* < 0.001*
Sex (%)				χ2 = 164.581,*P* < 0.001*
Male	8234(71.53)	7891(72.88)	343(50.07)	
female	3278(28.47)	2936(27.12)	342(49.93)	
Smoke				χ2 = 80.772, *P* < 0.001*
No	6768(58.79)	6253(57.75)	515(75.18)	
Yes	4744(41.21)	4574(42.25)	170(24.82)	
Thyroid nodule				χ2 = 9.764, *P* = 0.002*
No	5252(45.62)	4979(45.99)	273(39.85)	
Yes	6260(54.38)	5848(54.01)	412(60.15)	
Fatty liver				χ2 = 9.179, *P* = 0.002*
NO	4006(34.80)	3731(34.46)	275(40.15)	
Yes	7506(65.20)	7096(65.54)	410(59.85)	
Hypertension				χ2 = 0.224, *P* = 0.636
NO	7044(61.19)	6619(61.63)	425(62.04)	
Yes	4468(38.81)	4208(38.87)	260(37.96)	
SBP (mmHg)	121.53 ± 16.54	121.56 ± 16.48	121.36 ± 17.41	Z = 0.366, *P* = 0.714
DBP (mmHg)	79.49 ± 11.27	79.51 ± 11.27	79.22 ± 11.25	Z = 0.597, *P* = 0.550
BMI (kg/m^2^)	25.25 ± 3.29	25.28 ± 3.29	24.88 ± 3.24	Z = 2.914, *P* = 0.004*
HGB (g/L)	147.53 ± 14.73	147.79 ± 14.69	143.35 ± 14.71	Z = 8.264, *P* < 0.001*
TC (mmol/L)	4.70 ± 0.89	4.69 ± 0.89	4.87 ± 0.92	Z = 4.417, *P* < 0.001*
TG (mmol/L)	1.76 ± 1.38	1.75 ± 1.36	1.76 ± 1.75	Z = 0.627, *P* = 0.531
LDL-C (mmol/L)	3.03 ± 0.79	3.02 ± 0.79	3.13 ± 0.79	Z = 3.505, *P* < 0.001*
HDL-C (mmol/L)	1.25 ± 0.34	1.24 ± 0.34	1.29 ± 0.33	Z = 4.482, *P* < 0.001*
FBG (mmol/L)	5.67 ± 1.24	5.67 ± 1.25	5.56 ± 1.06	Z = 2.343, *P* = 0.019*
Hcy(μmol/L)	12.27 ± 7.70	12.29 ± 7.79	11.85 ± 5.90	Z = 2.409, *P* = 0.041*
UA(μmol/L)	345.97 ± 86.04	347.01 ± 85.91	329.54 ± 86.29	Z = 5.565，*P* < 0.001*
HbA1c(%)	5.77 ± 0.75	5.77 ± 0.76	5.75 ± 0.71	Z = 0.660, *P* = 0.509

*List of Abbreviations*: *SCH* subclinical hypothyroidism, *SBP* systolic blood pressure, *DBP* diastolic blood pressure, *BMI* body mass index, *HGB* hemoglobin determination, *TC* total cholesterol, *TG* triglyceride, *HDL-C* high density lipoprotein cholesterol, *LDL-C* low density lipoprotein cholesterol, *FBG* fasting blood glucose, *Hcy* homocysteine, *UA* blood uric acid, *HbA1c* hemoglobin A1c

*Significantly different between the two groups, (*P* < 0.05)

### Comparison of clinical indices between mild SCH and severe SCH

Of the 685 patients with SCH, 650 patients had mild SCH, while 35 patients had severe SCH, accounting for 5.11% of all SCH patients. A comparison of clinical indices between the control group, those with mild SCH, and those with severe SCH is shown in Table 2. By using the Kruskal-Wallis rank tests, there were statistically significant differences in age, sex distribution, smoking status, thyroid nodules, fatty liver, BMI, hemoglobin, TC, LDL-C, HDL-C and UA between the three groups (*P* < 0.05). However, there were no statistically significant differences in hypertension, TG, FBG, Hcy or HbA1c (*P* > 0.05). There were no statistically significant differences between the two groups (*P* > 0.05), when compared between the mild SCH subgroup and the severe SCH subgroup Table 2.

**Table 2:** Comparison of participant characteristics among the three groups (*N* = 11,512)

	Control group (***n*** = 10,827)	Mild SCH group (***n*** = 650)	Severe SCH group (***n*** = 35)	Statistical value ***P*** value
age(year)	48.94 ± 8.83	50.79 ± 9.52	51.49 ± 7.39	χ2 = 30.49, *P =* 0.0001 †
sex(%)				χ2 = 165.48, *P* < 0.001 †
male	7891(72.88)	323(49.69)	20(57.14)	
female	2936(27.12)	327(50.31)	15(42.86)	
Smoke				χ2 = 82.14, *P* < 0.001 †
No	6253(57.75)	492(75.69)	23(65.71)	
Yes	4574(42.25)	158(24.31)	12(34.29)	
Thyroid nodule				χ2 = 9.77, *P* = 0.008 †
No	4979(45.99)	259(39.85)	14(40.00)	
Yes	5848(54.01)	391(60.15)	21(60.00)	
Fatty liver				χ2 = 9.18, *P* = 0.010 †
NO	3731(34.46)	261(40.15)	14(40.00)	
Yes	7096(65.54)	389(59.85)	21(60.00)	
Hypertension				χ2 = 0.59, *P* = 0.742
NO	6619(61.63)	405(62.31)	20(57.14)	
Yes	4208(38.87)	245(37.69)	15(42.86)	
SBP(mmHg)	121.56 ± 16.48	121.40 ± 17.41	120.63 ± 19.87	χ2 = 0.25, *P* = 0.883
DBP(mmHg)	79.51 ± 11.27	79.26 ± 11.20	80.34 ± 12.11	χ2 = 1.24, *P* = 0.534
BMI (kg/m^2^)	25.28 ± 3.29	24.84 ± 3.18	25.52 ± 4.30	χ2 = 8.73, *P* = 0.013 †
HGB(g/L)	147.79 ± 14.69	143.09 ± 14.59	148.14 ± 16.22	χ2 = 71.58, *P* < 0.001 †
TC (mmol/L)	4.69 ± 0.89	4.86 ± 0.92	4.96 ± 0.94	χ2 = 19.59, *P* < 0.001 †
TG (mmol/L)	1.75 ± 1.36	1.77 ± 1.78	1.51 ± 0.86	χ2 = 1.17, *P* = 0.558
LDL-C(mmol/L)	3.02 ± 0.79	3.13 ± 0.79	3.26 ± 0.91	χ2 = 12.43, *P* = 0 .002 †
HDL-C(mmol/L)	1.24 ± 0.34	1.29 ± 0.33	1.36 ± 0.26	χ2 = 23.61, *P* < 0.001 †
FBG (mmol/L)	5.67 ± 1.25	5.56 ± 1.07	5.57 ± 0.83	χ2 = 5.88, *P* = 0.053
Hcy(μmol/L)	12.29 ± 7.79	11.765.74	13.338.21	χ2 = 4.88, *P* = 0.087
UA(μmol/L)	347.01 ± 85.91	329.69 ± 86.82	326.68 ± 76.82	χ2 = 30.99, *P* < 0.001 †
HbA1c(%)	5.77 ± 0.76	5.75 ± 0.72	5.74 ± 0.55	χ2 = 0.49, *P* = 0.781

There was no statistically significant difference between the mild SCH subgroup and the severe SCH subgroup((*P* > 0.05)

*List of Abbreviations*: *SCH* subclinical hypothyroidism, *SBP* systolic blood pressure, *DBP* diastolic blood pressure, *BMI* body mass index, *HGB* hemoglobin determination, *TC* total cholesterol, *TG* triglyceride, *HDL-C* high density lipoprotein cholesterol, *LDL-C* low density lipoprotein cholesterol, *FBG* fasting blood glucose, *Hcy* homocysteine, *UA* blood uric acid, *HbA1c* hemoglobin A1c

†: Significantly different among the three groups by the Kruskal-Wallis rank tests, (*P* < 0.05)

### Comparison of clinical indices between SCH patients and a strict control group

**Fig. 2:**
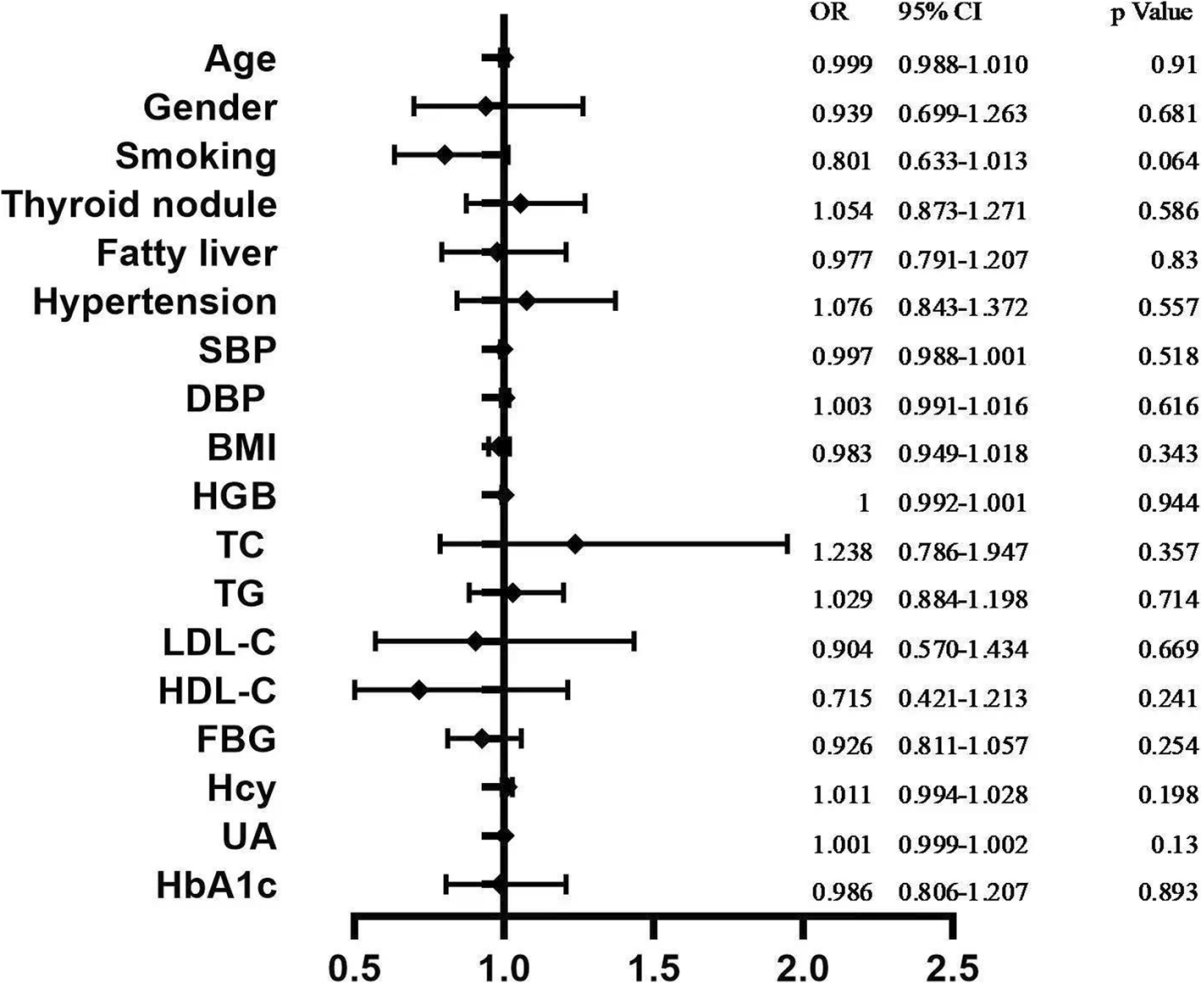
Results of multivariate logistics regression analysis (*N* = 2739). List of Abbreviations: OR: the Odds Ratio; CI: Confidence Intervals; SBP: systolic blood pressure; DBP: diastolic blood pressure; BMI: body mass index; HGB: hemoglobin determination; TC: total cholesterol; TG: triglyceride; HDL-C: high density lipoprotein cholesterol; LDL-C: low density lipoprotein cholesterol; FBG: fasting blood glucose; Hcy: homocysteine; UA: blood uric acid; HbA1c: hemoglobin A1c

Considering that differences in age and sex distribution may be serious confounding factors, the strict control group (*N* = 2054) was randomly selected from the 10,827 participants in the normal control group, matching the age and sex composition ratio in the SCH group. The sample size of the strict control group was approximately three times that of the SCH group. In addition, 2739 participants including a strict control group (*N* = 2054) and a SCH group (*N* = 685) were further studied. We found that age, sex distribution, smoking status, thyroid nodules, fatty liver, hypertension, BMI, hemoglobin, HDL-C, BFP, FBG, Hcy, and UA showed no statistically significant differences between the two groups (*P* < 0.05) (Table 3). Using SCH as a dependent variable and others as independent variables, a multivariate logistic regression analysis was performed. TC, TG, LDL-C, and HDL-C were not significant factors influencing SCH(*P* > 0.05), indicating that these parameters were not independent risk factors for SCH (Fig. 2).

**Table 3:** Recomparison of participant characteristics between SCH patients and the strict control group (*N* = 2739)

	Strict control group(***n*** = 2054)	SCH(***n*** = 685)	Statistical value, ***P*** value
Age(years)	50.84 ± 9.39	50.82 ± 9.42	*t* = 0.04, *P* = 0.966
Sex (%)			*χ*^2^ = 0.0001, *P* = 0.991
Male	1028(50.05)	343(50.07)	
female	1026(49.95)	342(49.93)	
Smoke			*χ*^2^ = 1.697, *P* = 0.193
No	1492(72.64)	515(75.18)	
Yes	562(27.36)	170(24.82)	
Thyroid nodule			*χ*^2^ = 0.253, *P* = 0.615
No	841(40.94)	273(39.85)	
Yes	1213(59.06)	412(60.15)	
Fatty liver			*χ*^2^ = 0.015, *P* = 0.903
NO	830(40.41)	275(40.15)	
Yes	1224(59.59)	410(59.85)	
Hypertension			*χ*^2^ = *0.291, P = 0.590*
NO	1298(63.19)	425(62.04)	
Yes	756(36.81)	26.(37.96)	
SBP (mmHg)	121.28 ± 17.26	121.36 ± 17.41	*Z* = 0.273, *P* = 0.785
DBP (mmHg)	78.72 ± 11.33	79.22 ± 11.25	*Z* = 1.026, *P* = 0.305
BMI (kg/m^2^)	24.85 ± 3.36	24.88 ± 3.24	*Z* = 0.478, *P* = 0.633
HGB (g/L)	143.01 ± 15.42	143.35 ± 14.71	*Z* = 0.277, *P* = 0.782
TC (mmol/L)	4.76 ± 0.88	4.87 ± 0.92	*Z* = 2.372, *P* = 0.018 *
TG (mmol/L)	1.59 ± 1.12	1.76 ± 1.75	*Z* = 2.385, *P* = 0.017 *
LDL-C (mmol/L)	3.07 ± 0.78	3.13 ± 0.79	*Z* = 2.008, *P* = 0.045 *
HDL-C (mmol/L)	1.31 ± 0.35	1.29 ± 0.33	*Z* = 0.763, *P* = 0.446
FBG (mmol/L)	5.64 ± 1.18	5.56 ± 1.06	*Z* = 1.340, *P* = 0.180
Hcy(μmol/L)	11.42 ± 5.53	11.85 ± 5.90	*Z* = 1.855, *P* = 0.634
UA(μmol/L)	321.81 ± 84.55	329.54 ± 86.29	*Z* = 2.031, *P* = 0.042 *
HbA1c(%)	5.79 ± 0.74	5.75 ± 0.71	*Z* = 0.797, *P* = 0.425

*List of Abbreviations*: *SCH* subclinical hypothyroidism, *SBP* systolic blood pressure, *DBP* diastolic blood pressure, *BMI* body mass index, *HGB* hemoglobin determination, *TC* total cholesterol, *TG* triglyceride, *HDL-C* high density lipoprotein cholesterol, *LDL-C* low density lipoprotein cholesterol, *FBG* fasting blood glucose, *Hcy* homocysteine, *UA* blood uric acid, *HbA1c* hemoglobin A1c

*Significantly different between the two groups, (*P* < 0.05)

To further analyze the impact of SCH severity on the above parameters, clinical indices were compared among the strict control group, the mild SCH subgroup and the severe SCH subgroup. Only TG showed a statistically significant difference among the three groups (*P <* 0.05) Table 4.

**Table 4:** Recomparison of participant characteristics among the three groups (*N* = 2739)

	Strict control group (***n*** = 2054)	Mild SCH (***n*** = 650)	Severe SCH (***n*** = 35)	Statistical value, ***P*** value
Age(years)	50.84 ± 9.39	50.79 ± 9.52	51.49 ± 7.39	χ2 = 0.158,*P* = 0.924
Sex (%)				χ2 = 0.553,*P* = 0.758
Male	1028(50.05)	323(49.69)	20(57.14)	
female	1026(49.95)	327(50.31)	15(42.86)	
Smoke				χ2 = 1.989,*P* = 0.370
No	1492(72.64)	492(75.69)	23(65.71)	
Yes	562(27.36)	158(24.31)	12(34.29)	
Thyroid nodule				χ2 = 0.184, *P* = 0.912
No	841(40.94)	259(39.85)	14(40.00)	
Yes	1213(59.06)	391(60.15)	21(60.00)	
Fatty liver				χ2 = 0.011, *P* = 0.995
NO	830(40.41)	261(40.15)	14(40.00)	
Yes	1224(59.59)	389(59.85)	21(60.00)	
Hypertension				χ2 = 0.469, *P* = 0.791
NO	1298(63.19)	405(62.31)	20(57.14)	
Yes	756(36.81)	245(37.69)	15(42.86)	
SBP (mmHg)	121.28 ± 17.26	121.40 ± 17.41	120.63 ± 19.87	χ2 = 0.180, *P* = 0.914
DBP (mmHg)	78.72 ± 11.33	79.26 ± 11.20	80.34 ± 12.11	χ2 = 1.916, *P* = 0.384
BMI (kg/m^2^)	24.85 ± 3.36	24.84 ± 3.18	25.52 ± 4.30	χ2 = 0.352, *P* = 0.838
HGB (g/L)	143.01 ± 15.42	143.09 ± 14.59	148.14 ± 16.22	χ2 = 2.598, *P* = 0.273
TC (mmol/L)	4.76 ± 0.88	4.86 ± 0.92	4.96 ± 0.94	χ2 = 5.711, *P* = 0.057
TG (mmol/L)	1.59 ± 1.12	1.77 ± 1.78	1.51 ± 0.86	χ2 = 6.527, *P* = 0.038 †
LDL-C (mmol/L)	3.07 ± 0.78	3.13 ± 0.79	3.26 ± 0.91	χ2 = 4.186, *P* = 0.123
HDL-C (mmol/L)	1.31 ± 0.35	1.29 ± 0.33	1.36 ± 0.26	χ2 = 3.919, *P* = 0.141
FBG (mmol/L)	5.64 ± 1.18	5.56 ± 1.07	5.57 ± 0.83	χ2 = 2.178, *P* = 0.337
Hcy(μmol/L)	11.42 ± 5.53	11.765.74	13.338.21	χ2 = 3.995, *P* = 0.136
UA(μmol/L)	321.81 ± 84.55	329.69 ± 86.82	326.68 ± 76.82	χ2 = 4.186, *P* = 0.123
HbA1c(%)	5.79 ± 0.74	5.75 ± 0.72	5.74 ± 0.55	χ2 = 0.703, *P* = 0.704

*List of Abbreviations*: *SCH* subclinical hypothyroidism, *SBP* systolic blood pressure, *DBP* diastolic blood pressure, *BMI* body mass index, *HGB* hemoglobin determination, *TC* total cholesterol, *TG* triglyceride, *HDL-C* high density lipoprotein cholesterol, *LDL-C* low density lipoprotein cholesterol, *FBG* fasting blood glucose, *Hcy* homocysteine, *UA* blood uric acid, *HbA1c* hemoglobin A1c

†: Significantly different among the three group by the Kruskal-Wallis rank tests, (*P* < 0.05)

## Discussion

SCH is an intermediate condition where those with normal thyroid function transition to hypothyroidism or those with hypothyroidism gradually recover to normal thyroid function. There are major controversies regarding the diagnosis and management of SCH [25, 26]. Although many studies have demonstrated that SCH has adverse effects on various physical functions, the diagnostic criteria for SCH are inconsistent, leaving the medical community perplexed [27]. Relaxing the diagnostic criteria will increase the number of SCH cases, but will also result in the misdiagnosis of genuine hypothyroidism cases as SCH. Our previous study compared five commonly used criteria for diagnosing SCH and found that elevated TSH but normal TT3, TT4, FT3, and FT4 concentrations might be the best criteria for SCH [4]. Since TT3, TT4, FT3, and FT4 were not detected simultaneously, many of the previously diagnosed cases of SCH might have reached the standard for hypothyroidism rather than SCH.

It is universally recognized that dyslipidemia can lead to the progression of atherosclerosis. Therefore, early detection of abnormal blood lipid levels and quick interventions are critical for the treatment of this disease and prevention of large-scale cardiovascular disease (CVD). Many studies have found that those with SCH are characterized by dyslipidemia, hyperglycemia, and cardiovascular dysfunction due to the lack of thyroid hormone. Some studies found an association between SCH and high TSH concentrations with all-cause mortality [28–30]. Nevertheless, it is debatable whether patients with SCH, whose thyroid hormones are within the normal range and have only elevated TSH, will experience the same outcomes [31]. For SCH, there are relatively few yet controversial studies on lipid abundance. In some studies, the total cholesterol level of SCH patients is higher than that in individuals with normal thyroid function, and the incidence of hypercholesterolemia is higher than that in normal individuals as well, findings that are positively correlated with the level of TSH [25, 32, 33]. Some studies have found that L-T4 replacement therapy can reduce serum total cholesterol and LDL-C levels in those with SCH [34–36]. However, recent guidelines strongly recommend against any hormone therapy for SCH [30]. Despite the lack of clinical symptoms in patients with SCH, it is recommended to opt for early prevention and treatment of ischemic heart disease.

There is an urgent need to identify individuals who are indeed in a state of SCH to accurately determine the physiological consequences of this disorder. In this study, only 685 individuals from 91,453 participants who finished the two physical checkups finally met the SCH standard. Although there were likely more than 685 patients with SCH among those people, these 685 subjects met the diagnostic criteria of SCH in each aspect. The same is true of the control group. Thus, we assume that the subjects of this study, both groups, underwent rigorous screening, which may reflect the true impact of SCH in the population.

When compared with the 10,827 participants in the control group, age, sex distribution, smoking status, thyroid nodules, fatty liver, hypertension, BMI, hemoglobin, TC, LDL-C, HDL-C, FBG, Hcy and UA in the SCH group were significantly different (*P* < 0.05). Stepwise logistic regression analysis demonstrated that sex, age, TC and body fat percentage were influential factors with statistical significance (*P* < 0.05), which is consistent with previous studies [13–15]. However, when the SCH group was divided into two subgroups (mild and severe), age, sex distribution, smoking status, thyroid nodule, fatty liver, hypertension, BMI, hemoglobin, TC, LDL-C, HDL-C, and UA were all significantly different between the three groups (*P* < 0.05).

The prevalence of SCH increases with age, and females are more prone to SCH, consistent with clinical characteristics [37]. Age and sex distribution may be significant confounding factors, which was why we matched age and sex distribution and established the strict control group. In further analysis, TC, TG, LDL-C and UA showed statistically significant differences between the SCH group and the strict control group (*P* < 0.05). However, multivariate logistic regression analysis indicated that no parameters including the above four factors were independent risk factors for SCH, inconsistent with previous studies [14–16]. Thus, it is suggested that the differences in TC, TG, LDL-C and UA between the two groups may be caused by other confounding factors.

Many factors can affect blood lipids. Theoretically, if there is a real correlation between two variables, the severity of the two variables is also related. However, we found that there was no statistically significant difference between the mild SCH subgroup and the severe SCH subgroup (*P* > 0.05), and only TG showed statistically significant difference among the strict control group, the mild SCH subgroup and the severe SCH subgroup, which suggested that SCH severity had no influence on the levels of TC, LDL-C or HDL-C, inconsistent with findings of other studies where the level of total cholesterol in SCH was positively correlated with the level of TSH [14, 15]. A meta-analysis reported that clinical parameters were more likely to be associated with thyroid hormone than with thyrotropin levels [38]. This may explain why there were no significant differences in various biochemical parameters between mild and severe cases of SCH in our study.

On the other hand, the clinical practice guidelines recommend observation rather than routine management of subclinical hypothyroidism because the benefits do not outweigh the risks. At the same time, our work indirectly suggests that in diagnosed SCH patients, and even in severe SCH patients, clinical follow-up and reexamination might be more important than an intervention before fully proving the definite relationship between blood lipids and SCH, especially among Chinese patients.

### Strengths and limitations

Our study had many strengths. First, to our knowledge, this was the initial study to evaluate the effects of SCH upon plasma lipid profile in the general Chinese population with rigorous diagnostic criteria. Second, the biological characterization was comprehensive, including age, sex, BMI, FBG, Hcy, UA, etc. Most importantly, the participants have a strict follow up and were re-examined more than 3 months apart (*N* = 91,453) (Fig. 1). Thirdly, the median interval between the two tests in the SCH group was 425 days, compared with 346 days for the control group. These indicators reflected the long-term status of subjects maintaining the SCH standard, confirming utility of the lipid profile data. Finally, we set up a specific strict control group according to the age and sex composition of SCH group. To some extent, this is the way to eliminate the above confounding factors to the greatest extent.

As one of the limitations of our study, our analysis involved predominantly those of Chinese heritage. Although we have included all available data, our results may not be applicable to other populations. Second, because physical examinations were opportunistic and optional, the loss of follow-up was more severe in this retrospective study. Relatively few subjects met the criteria for SCH, let alone severe SCH. The small sample size may affect the results of the statistical analysis. A long-term prospective cohort study is needed to confirm the effects of the transition from mild to severe SCH on blood lipids. For this reason, proactive countermeasures should be taken to include more subjects to confirm the variation in blood lipid levels in inpatients with SCH. Third, this study was limited to diagnosis without addressing the effect of interventions on SCH.

## Conclusions

Strict diagnostic criteria are essential for evaluating the effect of SCH on blood lipid changes. A final diagnosis of SCH should be established by initially elevated serum TSH with a reference range of TT3, TT4, FT3, and FT4, and the maintenance of these levels for more than 3 months. In our study, no definite association between SCH and blood lipid levels was finally confirmed, especially TC, LDL-C and HDL-C. SCH may have no relationship to the most concerning blood lipid profile. Although this view needs further confirmation, it could provide a new perspective to explain the debate on the hazard of SCH.

## Data Availability

The datasets used and/or analyzed during the current study are available from the corresponding author on reasonable request.

## Abbreviations

SCH: Subclinical hypothyroidism
SBP: Systolic blood pressure
DBP: Diastolic blood pressure
BMI: Body mass index
HGB: Hemoglobin determination
TC: Total cholesterol
TG: Triglyceride
HDL-C: High density lipoprotein cholesterol
LDL-C: Low density lipoprotein cholesterol
FBG: Fasting blood glucose
Hcy: Homocysteine
UA: Blood uric acid
HbA1c: Hemoglobin A1c
